# Association between *SUMF1* polymorphisms and COVID-19 severity

**DOI:** 10.1186/s12863-023-01133-6

**Published:** 2023-06-21

**Authors:** Shaohui Liang, Huixia Gao, Tongxin He, Li Li, Xin Zhang, Lei Zhao, Jie Chen, Yanyan Xie, Jie Bao, Yong Gao, Erhei Dai, Yuling Wang

**Affiliations:** 1 Department of Respiratory, Hebei Chest Hospital, Shijiazhuang, 050000 Hebei China; 2 Department of Laboratory Medicine, The Fifth Hospital of Shijiazhuang, Hebei Medical University, Shijiazhuang, 050021 Hebei China; 3 College of Plant Protection, Hunan Agricultural University, Changsha, 410128 Hunan China; 4 Intensive Care Unit, The Fifth Hospital of Shijiazhuang, Hebei Medical University, Shijiazhuang, 050021 Hebei China; 5 Department of Tuberculosis, The Fifth Hospital of Shijiazhuang, Hebei Medical University, Shijiazhuang, 050021 Hebei China; 6 The Second Internal Medicine, The Fifth Hospital of Shijiazhuang, Hebei Medical University, Shijiazhuang, 050021 Hebei China; 7 Graduate School of Hebei Medical University, Shijiazhuang, 050017 Hebei China

**Keywords:** COVID-19, Multiple sclerosis, *SUMF1*, rs794185

## Abstract

**Background:**

Evidence shows that genetic factors play important roles in the severity of coronavirus disease 2019 (COVID-19). Sulfatase modifying factor 1 (*SUMF1*) gene is involved in alveolar damage and systemic inflammatory response. Therefore, we speculate that it may play a key role in COVID-19.

**Results:**

We found that rs794185 was significantly associated with COVID-19 severity in Chinese population, under the additive model after adjusting for gender and age (for C allele = 0.62, 95% CI = 0.44–0.88, *P* = 0.0073, logistic regression). And this association was consistent with this in European population Genetics Of Mortality In Critical Care (GenOMICC: OR for C allele = 0.94, 95% CI = 0.90–0.98, *P* = 0.0037). Additionally, we also revealed a remarkable association between rs794185 and the prothrombin activity (PTA) in subjects (*P* = 0.015, Generalized Linear Model).

**Conclusions:**

In conclusion, our study for the first time identified that rs794185 in *SUMF1* gene was associated with the severity of COVID-19.

**Supplementary Information:**

The online version contains supplementary material available at 10.1186/s12863-023-01133-6.

## Introduction

The ongoing pandemic of coronavirus disease 2019 (COVID-19) caused by the severe acute respiratory syndrome coronavirus 2 (SARS-CoV-2) has made a serious public health threat worldwide, and has lasted for more than two years. So far, more than 570 million cases have been reported [1]. To date, many new SARS-CoV-2 variants have been observed, and these new variants have different epidemiological and biological characteristics [2–4]. The clinical spectrum of SARS-CoV-2 infection starts from mild influenza-like symptoms to severe pneumonia, and even acute respiratory distress syndrome (ARDS) and multiple organ failure [5–9]. The asymptomatic infection subjects and mild patients have a good prognosis after isolation [10, 11]. However, moderate, severe and critical patients still need special treatment, and the prognosis of severe and critical patients is poor, even leading to death [5, 12, 13]. Therefore, the study on the severity of COVID-19 is very important. One important line of research is the use of machine learning to understand and fight COVID-19. And this is currently an active research field. In many studies, machine learning has been proved to be very helpful in predicting the severity and mortality of COVID-19 [14–17]. It is beneficial to rational planning of medical resources. But only socio-demographic and presenting clinical data was used as input in most of the research on machine learning. Previous studies have shown that host genetic variation is related to the severity of COVID-19 [18–21]. And genetic determinism plays an important role in predicting the severity of COVID-19 and etiological guidance, which can provide a theoretical basis for individualized treatment of patients. The purpose of this study is to provide genetic ideas for predicting the severity of COVID-19.

Compared to other respiratory viruses, SARS-CoV-2 elicits a stronger, perduring, auto-aggressive inflammatory response [22], fueled by a massive cytokine release, causing coagulation dysfunction and multiple organ dysfunction syndrome in severe cases [23]. The occurrence and severity of COVID-19 largely depends on the host’s response to the infection, which echoes several aspects of Multiple sclerosis (MS) pathobiology. MS is a common neurological disease, which was caused by the failure of the immune system, characterized by persistent inflammation, demyelination and irreparable damage to the central nervous system [24, 25]. Like other autoimmune diseases, MS is associated with genetic factors [26]. Genome-wide association studies (GWASs) have observed a significant association between a single nucleotide polymorphism (SNP) rs794185 in sulfatase modifying factor 1 (*SUMF1*) gene and MS (*P* < 6.44 × 10^− 7^) [27]. *SUMF1* is biologically plausible for susceptibility to MS. For example, mutations in *SUMF1* could lead to multiple sulphatase deficiency and may indirectly regulate extracellular glutamate by altering the activity of steroid sulphatases, leading to neuroaxonal cell death, which is contributing aetiological factor in MS [28, 29]. Like immune disorder of MS, severe COVID-19 is also related to the “cytokine” storm caused by immune imbalance [30–32]. We speculated that COVID-19 and MS might have the same susceptibility gene. However, there are no studies showing a relationship between *SUMF1* gene and COVID-19. Here, we aimed to assess the possible association between *SUMF1* gene polymorphism (rs794185) and the severity of COVID-19.

## Results

### Characteristics of participants

The participants consisted of 285 cases and 141 controls, among which, 242 subjects were female, and 184 subjects were male. A significant difference was found in gender between the two groups (*P* = 0.010, Chi-square test). In addition, we also analyzed the difference of age, and it was significant between two groups (45.6 ± 18.0 vs. 29.8 ± 16.9, *P* < 0.001, Mann–Whitney U test). Compared to the individuals in control group, subjects from case group were older (Table 1). The laboratory indexes of case and control groups showed that white blood cell (WBC), neutrophil (NE), lymphocyte (LY), basophil (BAS), red blood cell (RBC), hemoglobin (HB), albumin (ALB), total bilirubin (TBIL), direct bilirubin (DBIL), fibrinogen (FIB), thrombin time (TT), erythrocyte sedimentation rate (ESR), C-reactive protein (CRP) and serum amyloid A (SAA) were significantly different between two groups (*P* < 0.05, Mann–Whitney U test) (Table 2).

**Table 1:** Clinical characteristics of the COVID-19 patients

Characteristics	Cases^a^ (*N* = 285)	Controls^b^ (*N* = 141)	Odds Ratio (95% CI)	*P*^*c*^ values
Gender, *n* (%)			0.545 (0.362–0.820)	0.003^d^
Male	109 (38.2)	75 (53.2)		
Female	176 (61.8)	66 (46.8)		
Age, Mean ± SD	45.6 ± 18.0	29.8 ± 16.9	1.050 (1.037–1.063)	< 0.001^e^

*CI* Confidence interval, *SD* Standard deviation

^a^Moderate, severe and critical patients were defined as cases

^b^Asymptomatic infection subjects were defined as controls

^c^*P* value < 0.05 indicates statistical significance

^d^Calculated by the Chi-square test

^e^Calculated by the Mann–Whitney U test

**Table 2:** Laboratory indexes of the study participants

Parameters	Cases^a^ (*N* = 285)	Controls^b^ (*N* = 141)	*Z*	*P*^c^ values
WBC (×10^9^/L)	6.048 ± 1.898	6.683 ± 2.068	-3.056	0.002
NE (×10^9^/L)	3.620 ± 1.429	4.602 ± 1.889	-2.021	0.043
LY (×10^9^/L)	1.405 ± 0.683	1.851 ± 0.538	-3.121	0.002
MONO (×10^9^/L)	0.339 ± 0.156	0.347 ± 0.126	-0.853	0.394
EOS (×10^9^/L)	0.046 ± 0.060	0.065 ± 0.058	-1.264	0.206
BAS (×10^9^/L)	0.017 ± 0.017	0.025 ± 0.014	-3.315	0.001
RBC (×10^12^/L)	4.398 ± 0.974	4.811 ± 0.606	-2.989	0.003
HB (g/L)	131.375 ± 29.748	144.500 ± 18.368	-3.261	0.001
PLT (×10^9^/L)	214.875 ± 83.806	247.518 ± 53.034	-0.775	0.438
ALB (g/L)	44.461 ± 5.609	49.261 ± 3.149	-6.897	< 0.001
ALT (U/L)	35.311 ± 49.912	29.313 ± 27.707	-1.45	0.147
AST (U/L)	32.732 ± 31.802	21.171 ± 6.054	-1.153	0.249
Scr (µmol/L)	66.829 ± 12.887	68.875 ± 12.651	-0.121	0.904
BUN (mmol/L)	4.820 ± 1.208	4.798 ± 1.246	-1.283	0.199
TBIL (µmol/L)	10.343 ± 6.128	11.614 ± 5.188	-2.026	0.043
DBIL (µmol/L)	3.334 ± 1.665	4.316 ± 2.259	-4.163	< 0.001
IBIL (µmol/L)	7.009 ± 4.798	7.298 ± 3.102	-0.983	0.326
D-dimer (µg/L)	330.688 ± 299.289	256.964 ± 300.139	-0.906	0.365
PT (s)	11.405 ± 2.186	11.421 ± 0.643	-0.644	0.520
INR	2.941 ± 14.431	0.993 ± 0.058	-0.41	0.681
PTA (%)	113.520 ± 8.446	104.079 ± 9.143	-0.221	0.825
APTT (s)	27.423 ± 4.373	27.082 ± 2.309	-0.474	0.635
FIB (g/L)	3.102 ± 0.859	2.425 ± 0.578	-4.577	< 0.001
TT (s)	16.763 ± 1.282	18.416 ± 5.985	-4.948	< 0.001
ESR (mm/h)	25.643 ± 26.681	11.821 ± 8.550	-2.919	0.004
CRP (mg/L)	9.672 ± 18.336	2.147 ± 12.127	-3.654	< 0.001
SAA (mg/L)	45.182 ± 86.163	12.886 ± 58.340	-5.634	< 0.001

*ALB* Albumin, *ALT* Alanine transaminase, *APTT* Activated partial thromboplastin time, *AST* Aspartate Aminotransferase, *BAS* Basophil, *BUN* Blood urea nitrogen, *CRP* C-reactive protein, *DBIL* Direct bilirubin, *EOS* Eosinophil, *ESR* Erythrocyte sedimentation rate, *FIB* Fibrinogen, *HB* Hemoglobin, *IBIL* Indirect bilirubin, *INR* International normalized ratio, *LY* Lymphocyt, *MONO* Monocyte, *NE* Neutrophil, *PLT* Platelet, *PT* Prothrombin time, *PTA* Prothrombin activity, *RBC* Red blood cell, *SAA* Serum amyloid A, *Scr* Serum creatinine, *TBIL* Total bilirubin, *TT* Thrombin time, *WBC* White blood cell

^a^Moderate, severe and critical patients were defined as cases. ^b^Asymptomatic infection subjects were defined as controls. ^c^*P* value < 0.05 indicates statistical significance and was calculated by the Mann–Whitney U test

### Association analysis between rs794185 and COVID-19 severity in the Chinese Han population and European population

Basic information of rs794185 in *SUMF1* gene was described in Table 4. In this study, rs794185 was genotyped using the Sequenom MassARRAY System. The calling rate of rs794185 was 99.77%. The Hardy-Weinberg equilibrium (HWE) tests in both case and control groups showed that SNP rs794185 could be subjected to further analysis (*P* > 0.05; Table 3).

**Table 3:** Hardy-Weinberg equilibrium tests for rs794185

SNP	Position	Gene	Allele1	Allele2	SNP calling	MAF	HWE-*P*^α^
rs794185	4,395,674	*SUMF1*	C	T	0.9977	0.3005	0.9084

*MAF* Minor allele frequency, *SNP* Single-nucleotide polymorphism, *SUMF1* Sulfatase modifying factor 1

*P* < 0.05 indicates statistical significance. ^α^HWE-P was calculated by the Hardy–Weinberg equilibrium (HWE) among the subjects

The genotype frequencies of rs794185 in control and case groups are shown in Table 4. Association analysis revealed that the risk of severe COVID-19 at the rs794185 site of the *SUMF1* gene was significantly reduced using TT genotype as a reference in the Chinese Han population under the additive model after adjusting for gender and age (odds ratio [OR] for C allele = 0.62, 95% CI = 0.44–0.88, *P* = 0.0073, logistic regression).

**Table 4:** Association between rs794185 and COVID-19 severity in the Chinese Han population

SNP	Model	Genotypes	Cases^a^ (*n*, %)	Controls^b^ (*n*, %)	Odds Ratio^c^ (95% CI)	*P*^d^ value
rs794185	Additive	CC	23 (8.1)	16 (11.3)	0.62 (0.44–0.88)	0.0073
		CT	113 (39.6)	65 (46.1)		
		TT	149 (52.3)	60 (42.5)		

*CI* confidence interval, *SNP* single-nucleotide polymorphism

^a^Moderate, severe and critical patients were defined as cases

^b^Asymptomatic infection subjects were defined as controls

^c^Odd Ratio was calculated for C allele

^d^*P* value < 0.05 indicates statistical significance and was calculated by logistic regression after adjusting for gender and age

After checking rs794185 in *SUMF1* gene in European population from an online public database Genetics Of Mortality In Critical Care (GenOMICC), we found that the result was consistent with this identified in the Chinese Han population. The risk of severe COVID-19 at the rs794185 site of the *SUMF1* gene was also significantly reduced in European population (GenOMICC: OR for C allele = 0.94, 95% CI = 0.90–0.98, *P* = 0.0037; Table 5).

**Table 5:** rs794185 in European population from online public resource

SNP	Study	Phenotype	Cases	Controls	Odds Ratio (95% CI)	*P*^a^ value
rs794185	GenOMICC (release 2)	Critical Covid cases vs. population controls	5986	42,845	0.94 (0.90–0.98)	0.0037

*CI* confidence interval, *GenOMICC* Genetics Of Mortality In Critical Care

^a^*P* value < 0.05 indicates statistical significance

### Stratification analyses: association between rs794185 and COVID-19 severity by gender or age

To eliminate potential confounding effects caused by age and gender, we further evaluated the alleles and COVID-19 severity stratified by age and gender (Table 6). The lower risk of severe COVID-19 was more evident among younger subjects (≤ 65 years old, OR = 0.72, 95% CI = 0.52-1.00, *P* = 0.0486, logistic regression) carrying the C allele. No apparent associations between rs794185 and COVID-19 severity were found in male and female (*P* > 0.05; Table 6).

**Table 6:** Stratification analyses: association between rs794185 and COVID-19 severity by gender or age

Variables	Allele	Cases^a^, *n* (%)	Controls^b^, *n* (%)	Odds Ratio (95% CI)	*P* values
Age (years)					
Age < 65	C	133 (27.3)	94 (34.1)	0.72 (0.52-1.00) 1.00	0.0486^c^
	T	355 (72.7)	182 (65.9)		
Age ≥ 65	C	26 (31.7)	3 (50.0)	0.47 (0.091–2.45) 1.00	0.3718^c^
	T	56 (68.3)	3 (50.0)		
Gender					
Male	C	59 (27.1)	52 (34.7)	0.67 (0.42–1.07) 1.00	0.0955^d^
	T	159 (72.9)	98 (65.3)		
Female	C	100 (28.4)	45 (34.1)	0.75 (0.48–1.15) 1.00	0.1856^d^
	T	252 (71.6)	87 (65.9)		

*CI* Confidence interval

*P* value < 0.05 indicates statistical significance

^a^Moderate, severe and critical patients were defined as cases

^b^Asymptomatic infection subjects were defined as controls

^c^Calculated by logistic regression adjusted for gender

^d^Calculated by logistic regression adjusted for age

### Association analyses between observed genotypes and clinical values

Association analyses between the observed genotypes and the clinical values were performed by GLM procedure (Table 7). The result revealed that there was a significant association between rs794185 and Prothrombin time activity (PTA) (*P* = 0.015, GLM).

**Table 7:** Association analyses between observed genotypes and clinical values

Parameters	CC	CT	TT	*P*^*a*^ values
WBC (×10^9^/L)	6.614 ± 0.317	6.174 ± 0.150	6.262 ± 0.138	0.455
NE (×10^9^/L)	3.958 ± 0.246	3.881 ± 0.117	3.927 ± 0.108	0.939
LY (×10^9^/L)	2.143 ± 0.132	1.852 ± 0.063	1.836 ± 0.058	0.097
MONO (×10^9^/L)	0.391 ± 0.023	0.358 ± 0.011	0.371 ± 0.010	0.372
EOS (×10^9^/L)	0.101 ± 0.017	0.081 ± 0.008	0.102 ± 0.008	0.146
BAS (×10^9^/L)	0.024 ± 0.003	0.026 ± 0.001	0.023 ± 0.001	0.223
RBC (×10^12^/L)	4.605 ± 0.103	4.546 ± 0.049	4.555 ± 0.045	0.874
HB (g/L)	135.513 ± 3.191	135.318 ± 1.515	135.000 ± 1.395	0.982
PLT (×10^9^/L)	250.513 ± 12.375	255.520 ± 5.875	250.554 ± 5.411	0.811
ALB (g/L)	44.575 ± 0.765	45.023 ± 0.369	45.108 ± 0.345	0.817
ALT (U/L)	32.005 ± 5.029	27.581 ± 2.418	31.768 ± 2.268	0.414
AST (U/L)	25.625 ± 2.592	23.475 ± 1.249	25.971 ± 1.169	0.331
Scr (µmol/L)	68.289 ± 2.798	68.478 ± 1.362	65.688 ± 1.269	0.296
BUN (mmol/L)	5.458 ± 0.236	5.255 ± 0.115	5.006 ± 0.107	0.115
TBIL (µmol/L)	9.933 ± 0.756	9.459 ± 0.364	10.040 ± 0.341	0.498
DBIL (µmol/L)	3.058 ± 0.296	2.966 ± 0.143	3.264 ± 0.133	0.306
IBIL (µmol/L)	6.875 ± 0.526	6.493 ± 0.253	6.776 ± 0.237	0.658
D-dimer (µg/L)	317.543 ± 177.696	423.433 ± 86.123	316.934 ± 77.712	0.636
PT (s)	11.397 ± 0.177	11.277 ± 0.089	11.423 ± 0.080	0.467
INR	0.965 ± 0.909	1.708 ± 0.458	0.995 ± 0.413	0.479
PTA (%)	122.074 ± 5.746	104.251 ± 2.892	104.455 ± 2.611	0.015
APTT (s)	27.363 ± 0.502	26.939 ± 0.253	27.032 ± 0.228	0.752
FIB (g/L)	2.846 ± 0.132	2.816 ± 0.067	2.782 ± 0.060	0.874
TT (s)	17.053 ± 0.423	17.201 ± 0.213	17.392 ± 0.192	0.681
ESR (mm/h)	16.550 ± 4.258	19.011 ± 1.985	18.765 ± 1.746	0.869
CRP (mg/L)	5.102 ± 1.900	4.187 ± 0.950	2.595 ± 0.863	0.313
SAA (mg/L)	34.374 ± 19.360	40.674 ± 10.681	18.883 ± 8.917	0.283

*ALB* albumin, *ALT* alanine transaminase, *APTT* activated partial thromboplastin time, *AST* aspartate Aminotransferase, *BAS* basophil, *BUN* blood urea nitrogen, *CRP* Creactive protein, *DBIL* direct bilirubin, *EOS *eosinophil, *ESR* erythrocyte sedimentation rate, *FIB* fibrinogen, *HB* hemoglobin, *IBIL* indirect bilirubin, *INR *international normalized ratio, *LY* lymphocyt, *MONO* monocyte, *NE* neutrophil, *PLT* platelet, *PT* prothrombin time, *PTA *prothrombin activity, *RBC* red blood cell, *SAA* serum amyloid A, *Scr* serum creatinine, *TBIL *total bilirubin, *TT *thrombin time, *WBC* white blood cell

^a^*P* values < 0.05 indicates statistical significance and were calculated by Generalized Linear Model (GLM) procedure

## Discussion

At present, the global epidemic continues. Studies have shown that age, sex, blood type, virulence of pathogens, and underlying diseases are related to the severity of COVID-19 [33–36]. But genetic factors of the individual also play an important role in the pathogenesis of COVID-19. So far, studies have reported that several polymorphisms are related to the susceptibility or severity of COVID-19, but these polymorphisms are different among different populations [37]. Obviously, the genetic determinant of COVID-19 severity is still unknown. The strong association with autoimmunity was peculiar of SARS-CoV-2 with respect to other Coronaviruses and respiratory viruses. Interestingly, MS-associated genes were mostly enriched in SARS-CoV-2 host’s interactors, suggesting pathophysiological overlaps that are worth investigating [38]. In particular, there are three pivotal crossroads of MS and COVID-19 immunological substrates: the type-1 IFN (IFN-I) response, the TH-17 axis, and the inflammasome pathway [39]. And a study by Moss et al. looked at the impact of the coronavirus pandemic on multiple sclerosis care at three centers, including the Cleveland Clinic, Johns Hopkins Hospital, and CEMCAT in Barcelona. The survey-based study surveyed 3028 patients with MS and found 77 (2.5%) suspected or confirmed cases of COVID-19. They found that these patients were more likely to know or live with COVID-19 patients [40]. These studies suggest us some genetic factors of COVID-19 largely overlap with MS. *SUMF1* gene polymorphism rs794185, as a genetic factor significantly related to autoimmune disease MS, has been confirmed by GWAS [27]. The possible reason is the deficiency of sulfatase caused by rs794185 variation. Therefore, we speculate that rs794185 is related to the severity of COVID-19.

So far, there are no studies showing the relationship between *SUMF1* gene and COVID-19 severity. But in our study, we found a significant association between rs794185 in *SUMF1* gene and COVID-19 severity (*P* = 0.0073). Subjects carrying the rs794185 locus C allele of the *SUMF1* gene had a lower risk of severe COVID-19. In Han Chinese subjects younger than 65 years of age, C allele carriers had a 0.72-fold reduced risk of severe COVID-19 compared to subjects with the T allele. And this significant association has been confirmed in European population in a COVID-19 GWAS online database. Also, according to the Genotype-Tissue Expression (GTEx) database (http://www.gtexportal.org/home/), expression quantitative trait loci (eQTL) analyses have indicated that rs794185 variant was associated with *SUMF1* gene expression in whole blood (*P* = 3.9 × 10^− 7^) [27]. Otherwise, several studies observed that *SUMF1* (-/-) mice developed emphysema-like phenotype following an arrest of alveolarization, and even systemic inflammation and neurodegeneration [41, 42]. Then, other studies showed that *SUMF1* gene variation increased the risk of Chronic Obstructive Pulmonary Disease (COPD) by affecting the expression, activity and localization in lung fibroblasts [43–45]. This suggests that the association between *SUMF1* and COVID-19 severity may be due to the following two factors.

On the one hand, *SUMF1* mutation causes pulmonary function decline by affecting alveolar function. Alveolar formation or alveolization is coordinated by fine regulation and complex interactions between growth factors and extracellular matrix proteins [46]. In the lung, glycosaminoglycans (GAGs) are dispersed in the extracellular matrix (ECM) [47]. Sulfatase activity requires a unique posttranslational modification, which is performed by *SUMF1* [48, 49], making it active to desulfate GAGs. It has been confirmed that sulfatase cannot be fully activated in *SUMF1* (-/-) mice. Highly sulfated GAG is deposited in the alveoli, which reduces the alveolar septum and increases the alveolar volume, resulting in decreased lung function [42]. Moreover, the role of GAGs in respiratory disease has been heightened by the current COVID-19 pandemic. GAGs are known to regulate growth factor distribution and activity according to their degree of sulfation. When *SUMF1* is mutated, highly sulfated GAGs promote growth factor *β* (TGF-*β*) signaling and the upregulation of TGF-*β* signaling in the lung has been observed in the *SUMF1* (-/-) mice. There is a developmental arrest in alveolar formation that reduces lung function. Bronchopulmonary dysplastic-like lungs due to suppression of alveolar septation were observed in transgenic mice with over expression of TGF-*β* between postnatal days 7 and 14 [50]. Similar results were obtained in neonatal rats overexpressing TGF-*β* [51]. And it has been confirmed by experiments in vivo injection of TGF-*β* neutralizing antibody leads to normalization of alveolarization [42]. These lung injuries have similarities with early-phase ARDS, a clinical manifestation in patients with severe COVID-19 [12, 52]. And some studies have shown that the level of serum TGF-*β* in severe COVID-19 group is significantly higher than that in the control group, which can predict the severe disease [53–55]. Also, application of TGF-*β* inhibitors will also relieve COVID-19 symptoms and sequelae [56–58]. At the same time, it also provides a new target for the treatment of lung tissue remodeling after COVID-19.

On the other hand, *SUMF1* gene mutations can cause a series of systemic responses. The mutations of *SUMF1* gene cause a decrease of sulfatase activity because of a post-translational modification defect [59]. And mammals have a single sulfate enzyme modification system. A team of researchers observed that in *SUMF1* (-/-) mice sulfatase activities were completely absent. All examined tissues showed progressive cell vacuolization and significant lysosomal storage of GAGs. And they detected a strong increase in the expression levels of inflammatory cytokines and of apoptotic markers in both the central nervous system and liver [41]. In the pathophysiology of ARDS induced by SARS-CoV-2, the overproduction of early response proinflammatory cytokines results in what has been described as a cytokine storm, leading to an increased risk of vascular hyperpermeability, multiorgan failure, and eventually death when the high cytokine concentrations are unabated over time [5]. It was demonstrated that the mutation of *SUMF1* gene caused systemic multisystem diseases including systemic inflammation, apoptosis and neurodegeneration. And this combined clinical symptom caused by sulfatase deficiency has also been observed in human individuals [60–62]. And the severity of the disease is related to the stability and residual activity of formylglycine generating enzyme (FGE) by the *SUMF1* gene encoding [63]. So, systemic multi-system dysfunction caused by *SUMF1* gene mutations may also promote the occurrence of severe COVID-19.

Additionally, activation of coagulation pathways during the immune response to SARS-CoV-2 infection results in overproduction of proinflammatory cytokines leading to multiorgan injury [5]. Our analyses revealed a remarkable association between rs794185 genotype and the PTA in patients (*P* = 0.015). This may be related to the coagulation dysfunction caused by COVID-19 [64, 65]. PTA is an important index reflecting liver coagulation function. Thrombin generation is tightly controlled by negative feedback loops and physiological anticoagulants, such as antithrombin III, tissue factor pathway inhibitor, and the protein C system [66]. During inflammation, all three of these control mechanisms can be impaired, with reduced anticoagulant concentrations due to reduced production and increasing consumption. This defective procoagulant–anticoagulant balance predisposes to the development of microthrombosis, disseminated intravascular coagulation, and multiorgan failure-evidenced in severe COVID-19 pneumonia with raised d-dimer concentrations being a poor prognostic feature and disseminated intravascular coagulation common in non-survivors [67, 68] So far, many studies have reported that COVID-19-induced coagulopathy (CIC) is commonly encountered [64, 69, 70]. While all coagulation parameters can be affected by COVID-19, there is considerable variability in the extent of these alterations and their correlation to disease severity and mortality [68, 71]. In addition to the impaired coagulation function caused by impaired liver function, it is speculated that the dysregulated immune responses orchestrated by inflammatory cytokines, lymphocyte cell death, hypoxia, and endothelial damage are involved [65]. Anyway, this finding contributes to early recognition of coagulation abnormalities among hospitalized COVID-19 patients.

Otherwise, our study has several limitations. Firstly, as a single-center study, the number of samples available is limited. In particular, the sample size of severe and critical patients in this study is small, while the proportion of severe and critical patients in European population is higher. So the verification about GenOMICC data is not very accurate. However, to some extent, it can reflect the relationship between *SUMF1* gene and the severity of COVID-19. In addition, it would be better to add other evaluation indicators for comprehensive analysis, such as lung function, lung CT.

## Conclusion

In summary, our study for the first time identified that rs794185 in *SUMF1* gene was associated with the severity of COVID-19. The risk of severe COVID-19 at the rs794185 site of the *SUMF1* gene was significantly reduced using TT genotype as a reference. This may be related to alveolar injury, systemic immune response and nervous system damage caused by infection.

This discovery might provide novel insights into the pathogenesis and clinical treatment of COVID-19. For clinicians, it can be used as a reference for predicting the severity of COVID-19. And it can help clinicians to plan medical resources effectively.

Of course, this result also needs larger sample size, multicenter research to systematically verify. In particular, it is necessary to expand the sample size of severe and critical patients.

## Methods

### Subject recruitment

In this study, we recruited 426 patients with SARS-CoV-2 infection in the Fifth Hospital of Shijiazhuang during January 2020 to May 2021, including 141 asymptomatic infection subjects, 261 moderate, 23 severe patients and 1 critical patient. The diagnosis of COVID-19 was made by a confirmed SARS-CoV-2 infection from nasopharyngeal swabs using real-time reverse transcriptase polymerase chain reaction (RT-PCR) assay. With reference to the Diagnosis and Treatment Protocol for COVID-19 (version 9.0), asymptomatic infection subjects were only positive for RT-PCR without any discomfort symptoms. According to ‘Diagnosis and Treatment Protocol for COVID-19’, the manifestation of “Moderate” stage included fever, respiratory symptoms and radiological evidence of pneumonia. The severe COVID-19 patients were those patients who had more severe clinical symptoms: respiratory distress, respiratory rate ≥ 30 beats/minute; means oxygen saturation ≤ 93% in a resting state; and arterial blood oxygen partial pressure/oxygen concentration ≤ 300 mmHg. In Critical stage, patients had at least one of the following symptoms: shock incidence; respiratory failure and requiring mechanical ventilation; and admission to intensive care unit (ICU) with other organ function failure. The classification of patients was based on the most serious classification during hospitalization. These assessments were done and checked by at least two experienced respiratory physicians. To identify the susceptibility loci contributing to severity of COVID-19, these subjects were divided into control group (asymptomatic infection subjects) and case group (hospitalized COVID-19 patients, i.e., moderate, severe and critical COVID-19 patients) according to the clinical characteristics. After obtaining the consent of the study participants, all information related to age, sex, laboratory indicators and disease types were recorded. The study was approved by the Medical Ethics Management Committee of the Fifth Hospital of Shijiazhuang (Ethics batch number: 202,230,714,010,258). Written informed consent was obtained from each participant. We confirmed that all methods were performed in accordance with the relevant guidelines and regulations.

### DNA extraction and genotyping

The whole blood samples of these patients (285 cases and 141 controls) were collected and cryopreserved with EDTA-containing tube at -80℃ until measurement. DNA was isolated according to the manufacturer’s instructions of Nucleic Acid Isolation or Purification Kit of DAAN GENE Company. The quantity and quality of the isolated genomic DNA were verified using two methods: (1) the DNA degradation and contamination were monitored on 1% agarose gels; and (2) the DNA concentration was measured using a Qubit 4.0 Fluorometer.

To genotype the SNP rs794185, we used the Sequenom MassARRAY System according to the manufacturer’s instructions (Sequenom) [72]. Briefly, locus-specific PCR and detection primers were designed using the MassARRAY Assay Design 3.0 software (Table 8). Approximate 15 ng of genomic DNAs for each sample were amplified by multiplex PCR, and the PCR products were then used for locus-specific single-base extension reactions. The resulting products were desalted and transferred to a 384-element SpectroCHIP array. Allele detection was performed using MALDI-TOF MS. The mass spectrograms were analyzed by the MassARRAY TYPER software (Sequenom). The cluster patterns of the genotyping data from Sequenom analyses were visually checked to confirm their good quality. For further quality control, 5% of the individuals in this study were randomly selected for repeated genotyping, and the results were 100% concordant.

**Table 8:** Primer sequences of rs794185

Gene	Sequences (5′-3′)
*SUMF1* (rs794185)	F - ACGTTGGATGCATGCCATTGTGGTGATTAC
	R - ACGTTGGATGCGTTTCTAATCTGGTGTGGG
	E - agGAAAACAGTCATTTCCTCCA

*E* Extended Primer Sequence, *F* Forward Primer Sequence, *R* Reverse Primer Sequence, *SUMF1* Sulfatase modifying factor 1

### Association analysis between *SUMF1* polymorphism and COVID-19 severity

The distribution frequencies of genotypes of rs794185 in the case group and the control group were analyzed in the Chinese Han population. After the Hardy-Weinberg equilibrium (HWE) test, the association analysis was carried out with PLINK software package (v1.9) [73]. We carried out SNP association analysis using logistic regression under an additive model with adjustment for age and gender.

To verify our result in European population, we checked an online public resource: Genetics Of Mortality In Critical Care (GenOMICC) (release 2) (an online public resource accessed from https://genomicc.org/data/) in 7,491 critically-ill cases with COVID-19 and 48,400 population controls. The associations were considered to be statistically significant when *P* < 0.05.

### Statistical analyses

Genotypes, clinical characteristics and laboratory indexes collected in Excel software were entered and then analyzed. The Chi-square test (χ^2^), Fisher’s exact test and Mann–Whitney U test were performed to compare the differences of clinical characteristics and laboratory indexes between the cases and controls using SPSS statistical software version 21.0. Generalized Linear Model (GLM) procedure was applied to test the association between observed genotypes and clinical values. Continuous and categorical variables were expressed as mean ± standard deviation and as percentages, respectively. Logistic regression was performed by using PLINK software package (v1.9). Significant level in all tests is considered 0.05.

## Supplementary Information

**Additional file 1: Table S1. **Genotypes of rs794185 in the Chinese Han population. **Table S2.** Snp rs794185 location information.

## Data Availability

All data generated or analysed during this study are included in this published article [and its Supplementary information files], the GenOMICC (release 2: 7491 critical Covid cases) (https://genomicc.org/data/) and the GTEx (release V8) websites (http://www.gtexportal.org/home/).

## Abbreviations

COVID-19: Coronavirus disease 2019
*SUMF1*: Sulfatase modifying factor 1
OR: Odds ratio
PTA: Prothrombin activity
GenOMICC: Genetics Of Mortality In Critical Care
SARS-CoV-2: Severe acute respiratory syndrome coronavirus 2
ARDS: Acute respiratory distress syndrome
MS: Multiple sclerosis
GWAS: Genome-wide association study
SNP: Single nucleotide polymorphism
CI: Confidence interval
SD: Standard deviation
ALB: Albumin
ALT: Alanine transaminase
APTT: Activated partial thromboplastin time
AST: Aspartate Aminotransferase
BAS: Basophil
BUN: Blood urea nitrogen
CRP: C-reactive protein
DBIL: Directbilirubin
EOS: Eosinophil
ESR: Erythrocyte sedimentation rate
FIB: Fibrinogen
HB: Hemoglobin
IBIL: Indirect bilirubin
INR: International normalized ratio
LY: Lymphocyte
MONO: Monocyte
NE: Neutrophil
PLT: Platelet
PT: Prothrombin time
RBC: Red blood cell
SAA: Serum amyloid A
Scr: Serum creatinine
TBIL: Total bilirubin
TT: Thrombintime
WBC: White blood cell.
MAF: Minor allele frequency
HWE: Hardy–Weinberg equilibrium
GLM: Generalized Linear Model
GTEx: Genotype-Tissue Expression
eQTL: Expression quantitative trait loci
COPD: ChronicObstructive Pulmonary Disease
GAGs: Glycosaminoglycans
ECM: Extracellular matrix
TGF-β: Transforming growth factorβ
FGE: Formylglycine generating enzyme
CIC: COVID-19-induced coagulopathy
RT-PCR: Real-timereverse transcriptase polymerase chain reactio
ICU: Intensive care unit
