# Supplementary material for: Association between SUMF1 polymorphisms and COVID-19 severity
Source: BMC Genom Data. 2023 Jun 21;24:34. doi: 10.1186/s12863-023-01133-6 (PMC10283310; doi:10.1186/s12863-023-01133-6)
Supplement: Supplementary file 1 — Additional file 1: Table S1. Genotypes of rs794185 in the Chinese Han population. Table S2. Snp rs794185 location information. [file 12863_2023_1133_MOESM1_ESM.docx]

**Table S1.** Genotypes of rs794185 in the Chinese Han population.

| Family ID | Individual ID | Paternal ID | Maternal ID | Sex | Phenotype | V_1_ | V_2_ |
| --- | --- | --- | --- | --- | --- | --- | --- |
| Y21070000478638 | Y21070000478638 | 0 | 0 | 0 | -9 | C | T |
| Y21070000478640 | Y21070000478640 | 0 | 0 | 0 | -9 | T | T |
| Y21070000478643 | Y21070000478643 | 0 | 0 | 0 | -9 | C | T |
| Y21070000478644 | Y21070000478644 | 0 | 0 | 0 | -9 | T | T |
| Y21070000478645 | Y21070000478645 | 0 | 0 | 0 | -9 | C | T |
| Y21070000478646 | Y21070000478646 | 0 | 0 | 0 | -9 | T | T |
| Y21070000478647 | Y21070000478647 | 0 | 0 | 0 | -9 | C | T |
| Y21070000478648 | Y21070000478648 | 0 | 0 | 0 | -9 | C | T |
| Y21070000478649 | Y21070000478649 | 0 | 0 | 0 | -9 | C | T |
| Y21070000478650 | Y21070000478650 | 0 | 0 | 0 | -9 | C | T |
| Y21070000478652 | Y21070000478652 | 0 | 0 | 0 | -9 | T | T |
| Y21070000478655 | Y21070000478655 | 0 | 0 | 0 | -9 | C | C |
| Y21070000478656 | Y21070000478656 | 0 | 0 | 0 | -9 | C | C |
| Y21070000478657 | Y21070000478657 | 0 | 0 | 0 | -9 | T | T |
| Y21070000478658 | Y21070000478658 | 0 | 0 | 0 | -9 | T | T |
| Y21070000478659 | Y21070000478659 | 0 | 0 | 0 | -9 | T | T |
| Y21070000478660 | Y21070000478660 | 0 | 0 | 0 | -9 | T | T |
| Y21070000478661 | Y21070000478661 | 0 | 0 | 0 | -9 | C | T |
| Y21070000478662 | Y21070000478662 | 0 | 0 | 0 | -9 | T | T |
| Y21070000478664 | Y21070000478664 | 0 | 0 | 0 | -9 | T | T |
| Y21070000478667 | Y21070000478667 | 0 | 0 | 0 | -9 | C | T |
| Y21070000478668 | Y21070000478668 | 0 | 0 | 0 | -9 | T | T |
| Y21070000478669 | Y21070000478669 | 0 | 0 | 0 | -9 | C | T |
| Y21070000478670 | Y21070000478670 | 0 | 0 | 0 | -9 | C | T |
| Y21070000478671 | Y21070000478671 | 0 | 0 | 0 | -9 | C | T |
| Y21070000478672 | Y21070000478672 | 0 | 0 | 0 | -9 | C | T |
| Y21070000478673 | Y21070000478673 | 0 | 0 | 0 | -9 | C | T |
| Y21070000478674 | Y21070000478674 | 0 | 0 | 0 | -9 | T | T |
| Y21070000478676 | Y21070000478676 | 0 | 0 | 0 | -9 | T | T |
| Y21070000478678 | Y21070000478678 | 0 | 0 | 0 | -9 | T | T |
| Y21070000478679 | Y21070000478679 | 0 | 0 | 0 | -9 | C | T |
| Y21070000478680 | Y21070000478680 | 0 | 0 | 0 | -9 | T | T |
| Y21070000478681 | Y21070000478681 | 0 | 0 | 0 | -9 | C | T |
| Y21070000478682 | Y21070000478682 | 0 | 0 | 0 | -9 | T | T |
| Y21070000478683 | Y21070000478683 | 0 | 0 | 0 | -9 | T | T |
| Y21070000478684 | Y21070000478684 | 0 | 0 | 0 | -9 | C | C |
| Y21070000478685 | Y21070000478685 | 0 | 0 | 0 | -9 | T | T |
| Y21070000478686 | Y21070000478686 | 0 | 0 | 0 | -9 | C | C |
| Y21070000478688 | Y21070000478688 | 0 | 0 | 0 | -9 | T | T |
| Y21070000478690 | Y21070000478690 | 0 | 0 | 0 | -9 | T | T |
| Y21070000478691 | Y21070000478691 | 0 | 0 | 0 | -9 | C | T |
| Y21070000478692 | Y21070000478692 | 0 | 0 | 0 | -9 | T | T |
| Y21070000478694 | Y21070000478694 | 0 | 0 | 0 | -9 | C | T |
| Y21070000478695 | Y21070000478695 | 0 | 0 | 0 | -9 | C | T |
| Y21070000478696 | Y21070000478696 | 0 | 0 | 0 | -9 | C | T |
| Y21070000478697 | Y21070000478697 | 0 | 0 | 0 | -9 | C | T |
| Y21070000478698 | Y21070000478698 | 0 | 0 | 0 | -9 | T | T |
| Y21070000478700 | Y21070000478700 | 0 | 0 | 0 | -9 | C | T |
| Y21070000478702 | Y21070000478702 | 0 | 0 | 0 | -9 | T | T |
| Y21070000478703 | Y21070000478703 | 0 | 0 | 0 | -9 | C | T |
| Y21070000478704 | Y21070000478704 | 0 | 0 | 0 | -9 | C | T |
| Y21070000478705 | Y21070000478705 | 0 | 0 | 0 | -9 | T | T |
| Y21070000478706 | Y21070000478706 | 0 | 0 | 0 | -9 | T | T |
| Y21070000478707 | Y21070000478707 | 0 | 0 | 0 | -9 | T | T |
| Y21070000478708 | Y21070000478708 | 0 | 0 | 0 | -9 | C | C |
| Y21070000478710 | Y21070000478710 | 0 | 0 | 0 | -9 | T | T |
| Y21070000478714 | Y21070000478714 | 0 | 0 | 0 | -9 | T | T |
| Y21070000478715 | Y21070000478715 | 0 | 0 | 0 | -9 | C | C |
| Y21070000478716 | Y21070000478716 | 0 | 0 | 0 | -9 | C | C |
| Y21070000478717 | Y21070000478717 | 0 | 0 | 0 | -9 | C | C |
| Y21070000478718 | Y21070000478718 | 0 | 0 | 0 | -9 | T | T |
| Y21070000478719 | Y21070000478719 | 0 | 0 | 0 | -9 | C | T |
| Y21070000478720 | Y21070000478720 | 0 | 0 | 0 | -9 | C | T |
| Y21070000478722 | Y21070000478722 | 0 | 0 | 0 | -9 | T | T |
| Y21070000478725 | Y21070000478725 | 0 | 0 | 0 | -9 | T | T |
| Y21070000478726 | Y21070000478726 | 0 | 0 | 0 | -9 | C | T |
| Y21070000478727 | Y21070000478727 | 0 | 0 | 0 | -9 | C | T |
| Y21070000478728 | Y21070000478728 | 0 | 0 | 0 | -9 | T | T |
| Y21070000478729 | Y21070000478729 | 0 | 0 | 0 | -9 | T | T |
| Y21070000478731 | Y21070000478731 | 0 | 0 | 0 | -9 | C | T |
| Y21070000478732 | Y21070000478732 | 0 | 0 | 0 | -9 | C | T |
| Y21070000478733 | Y21070000478733 | 0 | 0 | 0 | -9 | C | C |
| Y21070000478734 | Y21070000478734 | 0 | 0 | 0 | -9 | C | T |
| Y21070000478735 | Y21070000478735 | 0 | 0 | 0 | -9 | T | T |
| Y21070000478736 | Y21070000478736 | 0 | 0 | 0 | -9 | T | T |
| Y21070000478737 | Y21070000478737 | 0 | 0 | 0 | -9 | T | T |
| Y21070000478738 | Y21070000478738 | 0 | 0 | 0 | -9 | C | T |
| Y21070000478739 | Y21070000478739 | 0 | 0 | 0 | -9 | C | T |
| Y21070000478740 | Y21070000478740 | 0 | 0 | 0 | -9 | T | T |
| Y21070000478741 | Y21070000478741 | 0 | 0 | 0 | -9 | C | C |
| Y21070000478743 | Y21070000478743 | 0 | 0 | 0 | -9 | C | T |
| Y21070000478744 | Y21070000478744 | 0 | 0 | 0 | -9 | C | T |
| Y21070000478745 | Y21070000478745 | 0 | 0 | 0 | -9 | T | T |
| Y21070000478746 | Y21070000478746 | 0 | 0 | 0 | -9 | C | T |
| Y21070000478747 | Y21070000478747 | 0 | 0 | 0 | -9 | C | T |
| Y21070000478750 | Y21070000478750 | 0 | 0 | 0 | -9 | T | T |
| Y21070000478754 | Y21070000478754 | 0 | 0 | 0 | -9 | C | T |
| Y21070000478755 | Y21070000478755 | 0 | 0 | 0 | -9 | C | T |
| Y21070000478756 | Y21070000478756 | 0 | 0 | 0 | -9 | C | T |
| Y21070000478757 | Y21070000478757 | 0 | 0 | 0 | -9 | T | T |
| Y21070000478758 | Y21070000478758 | 0 | 0 | 0 | -9 | T | T |
| Y21070000478761 | Y21070000478761 | 0 | 0 | 0 | -9 | T | T |
| Y21070000478763 | Y21070000478763 | 0 | 0 | 0 | -9 | T | T |
| Y21070000478764 | Y21070000478764 | 0 | 0 | 0 | -9 | T | T |
| Y21070000478765 | Y21070000478765 | 0 | 0 | 0 | -9 | C | T |
| Y21070000478766 | Y21070000478766 | 0 | 0 | 0 | -9 | T | T |
| Y21070000478767 | Y21070000478767 | 0 | 0 | 0 | -9 | C | T |
| Y21070000478768 | Y21070000478768 | 0 | 0 | 0 | -9 | C | T |
| Y21070000478769 | Y21070000478769 | 0 | 0 | 0 | -9 | C | T |
| Y21070000478770 | Y21070000478770 | 0 | 0 | 0 | -9 | C | T |
| Y21070000478772 | Y21070000478772 | 0 | 0 | 0 | -9 | C | T |
| Y21070000478773 | Y21070000478773 | 0 | 0 | 0 | -9 | T | T |
| Y21070000478776 | Y21070000478776 | 0 | 0 | 0 | -9 | T | T |
| Y21070000478778 | Y21070000478778 | 0 | 0 | 0 | -9 | T | T |
| Y21070000478779 | Y21070000478779 | 0 | 0 | 0 | -9 | T | T |
| Y21070000478781 | Y21070000478781 | 0 | 0 | 0 | -9 | C | T |
| Y21070000478782 | Y21070000478782 | 0 | 0 | 0 | -9 | T | T |
| Y21070000478783 | Y21070000478783 | 0 | 0 | 0 | -9 | C | T |
| Y21070000478791 | Y21070000478791 | 0 | 0 | 0 | -9 | C | T |
| Y21070000478792 | Y21070000478792 | 0 | 0 | 0 | -9 | T | T |
| Y21070000478793 | Y21070000478793 | 0 | 0 | 0 | -9 | T | T |
| Y21070000478794 | Y21070000478794 | 0 | 0 | 0 | -9 | T | T |
| Y21070000478795 | Y21070000478795 | 0 | 0 | 0 | -9 | T | T |
| Y21070000478802 | Y21070000478802 | 0 | 0 | 0 | -9 | C | C |
| Y21070000478804 | Y21070000478804 | 0 | 0 | 0 | -9 | T | T |
| Y21070000478806 | Y21070000478806 | 0 | 0 | 0 | -9 | C | T |
| Y21070000478807 | Y21070000478807 | 0 | 0 | 0 | -9 | T | T |
| Y21070000478808 | Y21070000478808 | 0 | 0 | 0 | -9 | T | T |
| Y21070000478810 | Y21070000478810 | 0 | 0 | 0 | -9 | T | T |
| Y21070000478815 | Y21070000478815 | 0 | 0 | 0 | -9 | C | C |
| Y21070000478816 | Y21070000478816 | 0 | 0 | 0 | -9 | C | T |
| Y21070000478821 | Y21070000478821 | 0 | 0 | 0 | -9 | C | T |
| Y21070000478822 | Y21070000478822 | 0 | 0 | 0 | -9 | C | C |
| Y21070000478825 | Y21070000478825 | 0 | 0 | 0 | -9 | C | T |
| Y21070000478826 | Y21070000478826 | 0 | 0 | 0 | -9 | C | T |
| Y21070000478827 | Y21070000478827 | 0 | 0 | 0 | -9 | C | T |
| Y21070000478828 | Y21070000478828 | 0 | 0 | 0 | -9 | T | T |
| Y21070000478829 | Y21070000478829 | 0 | 0 | 0 | -9 | C | T |
| Y21070000478830 | Y21070000478830 | 0 | 0 | 0 | -9 | T | T |
| Y21070000478832 | Y21070000478832 | 0 | 0 | 0 | -9 | T | T |
| Y21070000478836 | Y21070000478836 | 0 | 0 | 0 | -9 | T | T |
| Y21070000478838 | Y21070000478838 | 0 | 0 | 0 | -9 | C | C |
| Y21070000478839 | Y21070000478839 | 0 | 0 | 0 | -9 | T | T |
| Y21070000478842 | Y21070000478842 | 0 | 0 | 0 | -9 | C | C |
| Y21070000478843 | Y21070000478843 | 0 | 0 | 0 | -9 | C | C |
| Y21070000478844 | Y21070000478844 | 0 | 0 | 0 | -9 | T | T |
| Y21070000478848 | Y21070000478848 | 0 | 0 | 0 | -9 | T | T |
| Y21070000478850 | Y21070000478850 | 0 | 0 | 0 | -9 | C | T |
| Y21070000478852 | Y21070000478852 | 0 | 0 | 0 | -9 | T | T |
| Y21070000478854 | Y21070000478854 | 0 | 0 | 0 | -9 | C | T |
| Y21070000478855 | Y21070000478855 | 0 | 0 | 0 | -9 | C | T |
| Y21070000478856 | Y21070000478856 | 0 | 0 | 0 | -9 | C | T |
| Y21070000478863 | Y21070000478863 | 0 | 0 | 0 | -9 | T | T |
| Y21070000478865 | Y21070000478865 | 0 | 0 | 0 | -9 | T | T |
| Y21070000478866 | Y21070000478866 | 0 | 0 | 0 | -9 | T | T |
| Y21070000478867 | Y21070000478867 | 0 | 0 | 0 | -9 | T | T |
| Y21070000478868 | Y21070000478868 | 0 | 0 | 0 | -9 | T | T |
| Y21070000478871 | Y21070000478871 | 0 | 0 | 0 | -9 | C | T |
| Y21070000478874 | Y21070000478874 | 0 | 0 | 0 | -9 | C | T |
| Y21070000478876 | Y21070000478876 | 0 | 0 | 0 | -9 | T | T |
| Y21070000478877 | Y21070000478877 | 0 | 0 | 0 | -9 | C | T |
| Y21070000478878 | Y21070000478878 | 0 | 0 | 0 | -9 | C | T |
| Y21070000478879 | Y21070000478879 | 0 | 0 | 0 | -9 | T | T |
| Y21070000478880 | Y21070000478880 | 0 | 0 | 0 | -9 | T | T |
| Y21070000478882 | Y21070000478882 | 0 | 0 | 0 | -9 | T | T |
| Y21070000478887 | Y21070000478887 | 0 | 0 | 0 | -9 | C | T |
| Y21070000478888 | Y21070000478888 | 0 | 0 | 0 | -9 | T | T |
| Y21070000478889 | Y21070000478889 | 0 | 0 | 0 | -9 | T | T |
| Y21070000478890 | Y21070000478890 | 0 | 0 | 0 | -9 | C | T |
| Y21070000478891 | Y21070000478891 | 0 | 0 | 0 | -9 | C | T |
| Y21070000478892 | Y21070000478892 | 0 | 0 | 0 | -9 | C | T |
| Y21070000478897 | Y21070000478897 | 0 | 0 | 0 | -9 | C | T |
| Y21070000478898 | Y21070000478898 | 0 | 0 | 0 | -9 | T | T |
| Y21070000478899 | Y21070000478899 | 0 | 0 | 0 | -9 | T | T |
| Y21070000478902 | Y21070000478902 | 0 | 0 | 0 | -9 | T | T |
| Y21070000478903 | Y21070000478903 | 0 | 0 | 0 | -9 | C | T |
| Y21070000478904 | Y21070000478904 | 0 | 0 | 0 | -9 | T | T |
| Y21070000478911 | Y21070000478911 | 0 | 0 | 0 | -9 | C | T |
| Y21070000478913 | Y21070000478913 | 0 | 0 | 0 | -9 | T | T |
| Y21070000478914 | Y21070000478914 | 0 | 0 | 0 | -9 | C | T |
| Y21070000478915 | Y21070000478915 | 0 | 0 | 0 | -9 | T | T |
| Y21070000478916 | Y21070000478916 | 0 | 0 | 0 | -9 | C | C |
| Y21070000478917 | Y21070000478917 | 0 | 0 | 0 | -9 | C | T |
| Y21070000478919 | Y21070000478919 | 0 | 0 | 0 | -9 | T | T |
| Y21070000478921 | Y21070000478921 | 0 | 0 | 0 | -9 | C | T |
| Y21070000478922 | Y21070000478922 | 0 | 0 | 0 | -9 | T | T |
| Y21070000478923 | Y21070000478923 | 0 | 0 | 0 | -9 | C | T |
| Y21070000478925 | Y21070000478925 | 0 | 0 | 0 | -9 | C | T |
| Y21070000478926 | Y21070000478926 | 0 | 0 | 0 | -9 | T | T |
| Y21070000478927 | Y21070000478927 | 0 | 0 | 0 | -9 | T | T |
| Y21070000478928 | Y21070000478928 | 0 | 0 | 0 | -9 | C | T |
| Y21070000478929 | Y21070000478929 | 0 | 0 | 0 | -9 | C | T |
| Y21070000478930 | Y21070000478930 | 0 | 0 | 0 | -9 | T | T |
| Y21070000478931 | Y21070000478931 | 0 | 0 | 0 | -9 | T | T |
| Y21070000478932 | Y21070000478932 | 0 | 0 | 0 | -9 | T | T |
| Y21070000478933 | Y21070000478933 | 0 | 0 | 0 | -9 | C | T |
| Y21070000478934 | Y21070000478934 | 0 | 0 | 0 | -9 | T | T |
| Y21070000478937 | Y21070000478937 | 0 | 0 | 0 | -9 | T | T |
| Y21070000478938 | Y21070000478938 | 0 | 0 | 0 | -9 | T | T |
| Y21070000478939 | Y21070000478939 | 0 | 0 | 0 | -9 | C | T |
| Y21070000478940 | Y21070000478940 | 0 | 0 | 0 | -9 | T | T |
| Y21070000478941 | Y21070000478941 | 0 | 0 | 0 | -9 | C | T |
| Y21070000478942 | Y21070000478942 | 0 | 0 | 0 | -9 | T | T |
| Y21070000478943 | Y21070000478943 | 0 | 0 | 0 | -9 | C | T |
| Y21070000478944 | Y21070000478944 | 0 | 0 | 0 | -9 | T | T |
| Y21070000478945 | Y21070000478945 | 0 | 0 | 0 | -9 | C | T |
| Y21070000478946 | Y21070000478946 | 0 | 0 | 0 | -9 | C | T |
| Y21070000478947 | Y21070000478947 | 0 | 0 | 0 | -9 | T | T |
| Y21070000478948 | Y21070000478948 | 0 | 0 | 0 | -9 | T | T |
| Y21070000478949 | Y21070000478949 | 0 | 0 | 0 | -9 | C | C |
| Y21070000478950 | Y21070000478950 | 0 | 0 | 0 | -9 | C | T |
| Y21070000478952 | Y21070000478952 | 0 | 0 | 0 | -9 | C | T |
| Y21070000478953 | Y21070000478953 | 0 | 0 | 0 | -9 | C | T |
| Y21070000478955 | Y21070000478955 | 0 | 0 | 0 | -9 | C | T |
| Y21070000478956 | Y21070000478956 | 0 | 0 | 0 | -9 | T | T |
| Y21070000478957 | Y21070000478957 | 0 | 0 | 0 | -9 | C | T |
| Y21070000478958 | Y21070000478958 | 0 | 0 | 0 | -9 | C | T |
| Y21070000478959 | Y21070000478959 | 0 | 0 | 0 | -9 | C | T |
| Y21070000478960 | Y21070000478960 | 0 | 0 | 0 | -9 | T | T |
| Y21070000478961 | Y21070000478961 | 0 | 0 | 0 | -9 | T | T |
| Y21070000478962 | Y21070000478962 | 0 | 0 | 0 | -9 | T | T |
| Y21070000478963 | Y21070000478963 | 0 | 0 | 0 | -9 | T | T |
| Y21070000478964 | Y21070000478964 | 0 | 0 | 0 | -9 | T | T |
| Y21070000478965 | Y21070000478965 | 0 | 0 | 0 | -9 | C | T |
| Y21070000478966 | Y21070000478966 | 0 | 0 | 0 | -9 | T | T |
| Y21070000478967 | Y21070000478967 | 0 | 0 | 0 | -9 | T | T |
| Y21070000478968 | Y21070000478968 | 0 | 0 | 0 | -9 | C | T |
| Y21070000478969 | Y21070000478969 | 0 | 0 | 0 | -9 | C | T |
| Y21070000478970 | Y21070000478970 | 0 | 0 | 0 | -9 | C | T |
| Y21070000478971 | Y21070000478971 | 0 | 0 | 0 | -9 | T | T |
| Y21070000478973 | Y21070000478973 | 0 | 0 | 0 | -9 | C | T |
| Y21070000478974 | Y21070000478974 | 0 | 0 | 0 | -9 | C | T |
| Y21070000478975 | Y21070000478975 | 0 | 0 | 0 | -9 | C | T |
| Y21070000478976 | Y21070000478976 | 0 | 0 | 0 | -9 | C | T |
| Y21070000478977 | Y21070000478977 | 0 | 0 | 0 | -9 | T | T |
| Y21070000478978 | Y21070000478978 | 0 | 0 | 0 | -9 | C | T |
| Y21070000478979 | Y21070000478979 | 0 | 0 | 0 | -9 | C | C |
| Y21070000478980 | Y21070000478980 | 0 | 0 | 0 | -9 | C | T |
| Y21070000478981 | Y21070000478981 | 0 | 0 | 0 | -9 | T | T |
| Y21070000478982 | Y21070000478982 | 0 | 0 | 0 | -9 | C | C |
| Y21070000478983 | Y21070000478983 | 0 | 0 | 0 | -9 | C | T |
| Y21070000478984 | Y21070000478984 | 0 | 0 | 0 | -9 | C | T |
| Y21070000478985 | Y21070000478985 | 0 | 0 | 0 | -9 | C | T |
| Y21070000478986 | Y21070000478986 | 0 | 0 | 0 | -9 | T | T |
| Y21070000478987 | Y21070000478987 | 0 | 0 | 0 | -9 | C | C |
| Y21070000478988 | Y21070000478988 | 0 | 0 | 0 | -9 | C | T |
| Y21070000478990 | Y21070000478990 | 0 | 0 | 0 | -9 | C | T |
| Y21070000478991 | Y21070000478991 | 0 | 0 | 0 | -9 | C | T |
| Y21070000478992 | Y21070000478992 | 0 | 0 | 0 | -9 | T | T |
| Y21070000478993 | Y21070000478993 | 0 | 0 | 0 | -9 | T | T |
| Y21070000478995 | Y21070000478995 | 0 | 0 | 0 | -9 | C | T |
| Y21070000478996 | Y21070000478996 | 0 | 0 | 0 | -9 | T | T |
| Y21070000478997 | Y21070000478997 | 0 | 0 | 0 | -9 | C | T |
| Y21070000478999 | Y21070000478999 | 0 | 0 | 0 | -9 | C | T |
| Y21070000479000 | Y21070000479000 | 0 | 0 | 0 | -9 | C | T |
| Y21070000479002 | Y21070000479002 | 0 | 0 | 0 | -9 | T | T |
| Y21070000479003 | Y21070000479003 | 0 | 0 | 0 | -9 | T | T |
| Y21070000479004 | Y21070000479004 | 0 | 0 | 0 | -9 | C | C |
| Y21070000479005 | Y21070000479005 | 0 | 0 | 0 | -9 | C | T |
| Y21070000479006 | Y21070000479006 | 0 | 0 | 0 | -9 | C | T |
| Y21070000479008 | Y21070000479008 | 0 | 0 | 0 | -9 | T | T |
| Y21070000479009 | Y21070000479009 | 0 | 0 | 0 | -9 | T | T |
| Y21070000479010 | Y21070000479010 | 0 | 0 | 0 | -9 | C | T |
| Y21070000479011 | Y21070000479011 | 0 | 0 | 0 | -9 | T | T |
| Y21070000479012 | Y21070000479012 | 0 | 0 | 0 | -9 | C | T |
| Y21070000479014 | Y21070000479014 | 0 | 0 | 0 | -9 | C | C |
| Y21070000479015 | Y21070000479015 | 0 | 0 | 0 | -9 | T | T |
| Y21070000479016 | Y21070000479016 | 0 | 0 | 0 | -9 | C | T |
| Y21070000479017 | Y21070000479017 | 0 | 0 | 0 | -9 | T | T |
| Y21070000479018 | Y21070000479018 | 0 | 0 | 0 | -9 | T | T |
| Y21070000479019 | Y21070000479019 | 0 | 0 | 0 | -9 | C | T |
| Y21070000479020 | Y21070000479020 | 0 | 0 | 0 | -9 | T | T |
| Y21070000479021 | Y21070000479021 | 0 | 0 | 0 | -9 | T | T |
| Y21070000479022 | Y21070000479022 | 0 | 0 | 0 | -9 | T | T |
| Y21070000479023 | Y21070000479023 | 0 | 0 | 0 | -9 | C | T |
| Y21070000479024 | Y21070000479024 | 0 | 0 | 0 | -9 | C | T |
| Y21070000479025 | Y21070000479025 | 0 | 0 | 0 | -9 | C | T |
| Y21070000479026 | Y21070000479026 | 0 | 0 | 0 | -9 | C | C |
| Y21070000479027 | Y21070000479027 | 0 | 0 | 0 | -9 | C | T |
| Y21070000479028 | Y21070000479028 | 0 | 0 | 0 | -9 | T | T |
| Y21070000479029 | Y21070000479029 | 0 | 0 | 0 | -9 | C | T |
| Y21070000479030 | Y21070000479030 | 0 | 0 | 0 | -9 | T | T |
| Y21070000479031 | Y21070000479031 | 0 | 0 | 0 | -9 | T | T |
| Y21070000479033 | Y21070000479033 | 0 | 0 | 0 | -9 | T | T |
| Y21070000479034 | Y21070000479034 | 0 | 0 | 0 | -9 | T | T |
| Y21070000479035 | Y21070000479035 | 0 | 0 | 0 | -9 | T | T |
| Y21070000479036 | Y21070000479036 | 0 | 0 | 0 | -9 | T | T |
| Y21070000479037 | Y21070000479037 | 0 | 0 | 0 | -9 | C | C |
| Y21070000479038 | Y21070000479038 | 0 | 0 | 0 | -9 | C | T |
| Y21070000479039 | Y21070000479039 | 0 | 0 | 0 | -9 | T | T |
| Y21070000479040 | Y21070000479040 | 0 | 0 | 0 | -9 | C | T |
| Y21070000479041 | Y21070000479041 | 0 | 0 | 0 | -9 | T | T |
| Y21070000479042 | Y21070000479042 | 0 | 0 | 0 | -9 | T | T |
| Y21070000479043 | Y21070000479043 | 0 | 0 | 0 | -9 | C | T |
| Y21070000479044 | Y21070000479044 | 0 | 0 | 0 | -9 | C | T |
| Y21070000479045 | Y21070000479045 | 0 | 0 | 0 | -9 | C | T |
| Y21070000479046 | Y21070000479046 | 0 | 0 | 0 | -9 | T | T |
| Y21070000479047 | Y21070000479047 | 0 | 0 | 0 | -9 | T | T |
| Y21070000479048 | Y21070000479048 | 0 | 0 | 0 | -9 | C | T |
| Y21070000479049 | Y21070000479049 | 0 | 0 | 0 | -9 | T | T |
| Y21070000479050 | Y21070000479050 | 0 | 0 | 0 | -9 | C | T |
| Y21070000479052 | Y21070000479052 | 0 | 0 | 0 | -9 | C | T |
| Y21070000479054 | Y21070000479054 | 0 | 0 | 0 | -9 | C | T |
| Y21070000479055 | Y21070000479055 | 0 | 0 | 0 | -9 | T | T |
| Y21070000479056 | Y21070000479056 | 0 | 0 | 0 | -9 | C | C |
| Y21070000479057 | Y21070000479057 | 0 | 0 | 0 | -9 | T | T |
| Y21070000479058 | Y21070000479058 | 0 | 0 | 0 | -9 | T | T |
| Y21070000479059 | Y21070000479059 | 0 | 0 | 0 | -9 | T | T |
| Y21070000479060 | Y21070000479060 | 0 | 0 | 0 | -9 | T | T |
| Y21070000479061 | Y21070000479061 | 0 | 0 | 0 | -9 | C | T |
| Y21070000479062 | Y21070000479062 | 0 | 0 | 0 | -9 | T | T |
| Y21070000479063 | Y21070000479063 | 0 | 0 | 0 | -9 | C | T |
| Y21070000479064 | Y21070000479064 | 0 | 0 | 0 | -9 | T | T |
| Y21070000479065 | Y21070000479065 | 0 | 0 | 0 | -9 | C | T |
| Y21070000479066 | Y21070000479066 | 0 | 0 | 0 | -9 | C | T |
| Y21070000479067 | Y21070000479067 | 0 | 0 | 0 | -9 | T | T |
| Y21070000479068 | Y21070000479068 | 0 | 0 | 0 | -9 | T | T |
| Y21070000479069 | Y21070000479069 | 0 | 0 | 0 | -9 | C | C |
| Y21070000479071 | Y21070000479071 | 0 | 0 | 0 | -9 | C | T |
| Y21070000479072 | Y21070000479072 | 0 | 0 | 0 | -9 | C | C |
| Y21070000479073 | Y21070000479073 | 0 | 0 | 0 | -9 | C | T |
| Y21070000479074 | Y21070000479074 | 0 | 0 | 0 | -9 | C | T |
| Y21070000479075 | Y21070000479075 | 0 | 0 | 0 | -9 | C | T |
| Y21070000479076 | Y21070000479076 | 0 | 0 | 0 | -9 | C | C |
| Y21070000479079 | Y21070000479079 | 0 | 0 | 0 | -9 | T | T |
| Y21070000479080 | Y21070000479080 | 0 | 0 | 0 | -9 | T | T |
| Y21070000479081 | Y21070000479081 | 0 | 0 | 0 | -9 | T | T |
| Y21070000479083 | Y21070000479083 | 0 | 0 | 0 | -9 | T | T |
| Y21070000479084 | Y21070000479084 | 0 | 0 | 0 | -9 | T | T |
| Y21070000479085 | Y21070000479085 | 0 | 0 | 0 | -9 | T | T |
| Y21070000479086 | Y21070000479086 | 0 | 0 | 0 | -9 | C | T |
| Y21070000479087 | Y21070000479087 | 0 | 0 | 0 | -9 | T | T |
| Y21070000479088 | Y21070000479088 | 0 | 0 | 0 | -9 | C | T |
| Y21070000479090 | Y21070000479090 | 0 | 0 | 0 | -9 | C | T |
| Y21070000479091 | Y21070000479091 | 0 | 0 | 0 | -9 | C | T |
| Y21070000479092 | Y21070000479092 | 0 | 0 | 0 | -9 | C | T |
| Y21070000479093 | Y21070000479093 | 0 | 0 | 0 | -9 | T | T |
| Y21070000479094 | Y21070000479094 | 0 | 0 | 0 | -9 | T | T |
| Y21070000479095 | Y21070000479095 | 0 | 0 | 0 | -9 | T | T |
| Y21070000479097 | Y21070000479097 | 0 | 0 | 0 | -9 | T | T |
| Y21070000479098 | Y21070000479098 | 0 | 0 | 0 | -9 | T | T |
| Y21070000479099 | Y21070000479099 | 0 | 0 | 0 | -9 | C | T |
| Y21070000479100 | Y21070000479100 | 0 | 0 | 0 | -9 | C | T |
| Y21070000479101 | Y21070000479101 | 0 | 0 | 0 | -9 | C | C |
| Y21070000479102 | Y21070000479102 | 0 | 0 | 0 | -9 | C | T |
| Y21070000479103 | Y21070000479103 | 0 | 0 | 0 | -9 | T | T |
| Y21070000479104 | Y21070000479104 | 0 | 0 | 0 | -9 | T | T |
| Y21070000479105 | Y21070000479105 | 0 | 0 | 0 | -9 | T | T |
| Y21070000479109 | Y21070000479109 | 0 | 0 | 0 | -9 | C | T |
| Y21070000479110 | Y21070000479110 | 0 | 0 | 0 | -9 | T | T |
| Y21070000479112 | Y21070000479112 | 0 | 0 | 0 | -9 | C | T |
| Y21070000479113 | Y21070000479113 | 0 | 0 | 0 | -9 | C | C |
| Y21070000479114 | Y21070000479114 | 0 | 0 | 0 | -9 | C | T |
| Y21070000479115 | Y21070000479115 | 0 | 0 | 0 | -9 | T | T |
| Y21070000479116 | Y21070000479116 | 0 | 0 | 0 | -9 | C | T |
| Y21070000479117 | Y21070000479117 | 0 | 0 | 0 | -9 | T | T |
| Y21070000479118 | Y21070000479118 | 0 | 0 | 0 | -9 | T | T |
| Y21070000479119 | Y21070000479119 | 0 | 0 | 0 | -9 | C | T |
| Y21070000479120 | Y21070000479120 | 0 | 0 | 0 | -9 | C | C |
| Y21070000479121 | Y21070000479121 | 0 | 0 | 0 | -9 | T | T |
| Y21070000479123 | Y21070000479123 | 0 | 0 | 0 | -9 | C | T |
| Y21070000479124 | Y21070000479124 | 0 | 0 | 0 | -9 | T | T |
| Y21070000479125 | Y21070000479125 | 0 | 0 | 0 | -9 | C | C |
| Y21070000479127 | Y21070000479127 | 0 | 0 | 0 | -9 | T | T |
| Y21070000479128 | Y21070000479128 | 0 | 0 | 0 | -9 | T | T |
| Y21070000479129 | Y21070000479129 | 0 | 0 | 0 | -9 | T | T |
| Y21070000479132 | Y21070000479132 | 0 | 0 | 0 | -9 | T | T |
| Y21070000479134 | Y21070000479134 | 0 | 0 | 0 | -9 | T | T |
| Y21070000479135 | Y21070000479135 | 0 | 0 | 0 | -9 | T | T |
| Y21070000479136 | Y21070000479136 | 0 | 0 | 0 | -9 | C | T |
| Y21070000479137 | Y21070000479137 | 0 | 0 | 0 | -9 | T | T |
| Y21070000479140 | Y21070000479140 | 0 | 0 | 0 | -9 | C | T |
| Y21070000479142 | Y21070000479142 | 0 | 0 | 0 | -9 | T | T |
| Y21070000479143 | Y21070000479143 | 0 | 0 | 0 | -9 | T | T |
| Y21070000479144 | Y21070000479144 | 0 | 0 | 0 | -9 | C | T |
| Y21070000479146 | Y21070000479146 | 0 | 0 | 0 | -9 | T | T |
| Y21070000479147 | Y21070000479147 | 0 | 0 | 0 | -9 | T | T |
| Y21070000479148 | Y21070000479148 | 0 | 0 | 0 | -9 | C | T |
| Y21070000479150 | Y21070000479150 | 0 | 0 | 0 | -9 | T | T |
| Y21070000479151 | Y21070000479151 | 0 | 0 | 0 | -9 | T | T |
| Y21070000479154 | Y21070000479154 | 0 | 0 | 0 | -9 | T | T |
| Y21070000479156 | Y21070000479156 | 0 | 0 | 0 | -9 | T | T |
| Y21070000479157 | Y21070000479157 | 0 | 0 | 0 | -9 | C | C |
| Y21070000479158 | Y21070000479158 | 0 | 0 | 0 | -9 | T | T |
| Y21070000479159 | Y21070000479159 | 0 | 0 | 0 | -9 | C | C |
| Y21070000479161 | Y21070000479161 | 0 | 0 | 0 | -9 | C | C |
| Y21070000479162 | Y21070000479162 | 0 | 0 | 0 | -9 | T | T |
| Y21070000479163 | Y21070000479163 | 0 | 0 | 0 | -9 | T | T |
| Y21070000479164 | Y21070000479164 | 0 | 0 | 0 | -9 | C | T |
| Y21070000479165 | Y21070000479165 | 0 | 0 | 0 | -9 | T | T |
| Y21070000479171 | Y21070000479171 | 0 | 0 | 0 | -9 | C | T |
| Y21070000479172 | Y21070000479172 | 0 | 0 | 0 | -9 | C | T |
| Y21070000479173 | Y21070000479173 | 0 | 0 | 0 | -9 | C | C |
| Y21070000479175 | Y21070000479175 | 0 | 0 | 0 | -9 | C | T |
| Y21070000479176 | Y21070000479176 | 0 | 0 | 0 | -9 | C | T |
| Y21070000479177 | Y21070000479177 | 0 | 0 | 0 | -9 | T | T |
| Y21070000479178 | Y21070000479178 | 0 | 0 | 0 | -9 | T | T |
| Y21100000493870 | Y21100000493870 | 0 | 0 | 0 | -9 | T | T |
| Y21100000493871 | Y21100000493871 | 0 | 0 | 0 | -9 | C | T |
| Y21100000493872 | Y21100000493872 | 0 | 0 | 0 | -9 | T | T |
| Y21100000493874 | Y21100000493874 | 0 | 0 | 0 | -9 | T | T |
| Y21100000493875 | Y21100000493875 | 0 | 0 | 0 | -9 | T | T |
| Y21100000493877 | Y21100000493877 | 0 | 0 | 0 | -9 | T | T |
| Y21100000493878 | Y21100000493878 | 0 | 0 | 0 | -9 | T | T |
| Y21100000493879 | Y21100000493879 | 0 | 0 | 0 | -9 | T | T |
| Y21100000493880 | Y21100000493880 | 0 | 0 | 0 | -9 | C | T |
| Y21100000493881 | Y21100000493881 | 0 | 0 | 0 | -9 | T | T |
| Y21100000493882 | Y21100000493882 | 0 | 0 | 0 | -9 | C | C |
| Y21100000493883 | Y21100000493883 | 0 | 0 | 0 | -9 | C | T |
| Y21100000493884 | Y21100000493884 | 0 | 0 | 0 | -9 | C | C |
| Y21100000493885 | Y21100000493885 | 0 | 0 | 0 | -9 | T | T |
| Y21100000493886 | Y21100000493886 | 0 | 0 | 0 | -9 | T | T |
| Y21100000493887 | Y21100000493887 | 0 | 0 | 0 | -9 | T | T |
| Y21100000493888 | Y21100000493888 | 0 | 0 | 0 | -9 | C | T |
| Y21100000493889 | Y21100000493889 | 0 | 0 | 0 | -9 | C | T |
| Y21100000493890 | Y21100000493890 | 0 | 0 | 0 | -9 | T | T |
| Y21100000493891 | Y21100000493891 | 0 | 0 | 0 | -9 | C | T |
| Y21100000493892 | Y21100000493892 | 0 | 0 | 0 | -9 | T | T |
| Y21100000493893 | Y21100000493893 | 0 | 0 | 0 | -9 | C | T |
| Y21100000493894 | Y21100000493894 | 0 | 0 | 0 | -9 | C | T |
| Y21100000493896 | Y21100000493896 | 0 | 0 | 0 | -9 | T | T |
| Y21100000493897 | Y21100000493897 | 0 | 0 | 0 | -9 | T | T |
| Y21100000493899 | Y21100000493899 | 0 | 0 | 0 | -9 | C | T |
| Y21100000493902 | Y21100000493902 | 0 | 0 | 0 | -9 | C | T |
| Y21100000493903 | Y21100000493903 | 0 | 0 | 0 | -9 | T | T |
| Y21100000493904 | Y21100000493904 | 0 | 0 | 0 | -9 | C | T |
| Y21100000493905 | Y21100000493905 | 0 | 0 | 0 | -9 | T | T |
| Y21100000493906 | Y21100000493906 | 0 | 0 | 0 | -9 | T | T |
| Y21100000493907 | Y21100000493907 | 0 | 0 | 0 | -9 | C | T |
| Y21100000493909 | Y21100000493909 | 0 | 0 | 0 | -9 | C | T |
| Y21100000493911 | Y21100000493911 | 0 | 0 | 0 | -9 | T | T |
| Y21100000493912 | Y21100000493912 | 0 | 0 | 0 | -9 | T | T |
| Y21100000493913 | Y21100000493913 | 0 | 0 | 0 | -9 | C | T |
| Y21100000493914 | Y21100000493914 | 0 | 0 | 0 | -9 | C | T |
| Y21100000493915 | Y21100000493915 | 0 | 0 | 0 | -9 | C | T |
| Y21100000493916 | Y21100000493916 | 0 | 0 | 0 | -9 | T | T |

**Table S2.** Snp rs794185 location information.

| Chromosome | Marker ID | Genetic distance | Physical position |
| --- | --- | --- | --- |
| 3 | rs794185 | 0 | 4395674 |
